# Editorial

**DOI:** 10.1155/S1463924683000188

**Published:** 1983


Journal of Automatic Chemistry, Volume 5, Number 2 (April–June 1983), pages 68–70

### Editorial

### Introduction to the papers presented at the symposium on cost analysis held on 22 April 1982 in Barcelona during the First International Congress on Automation in Clinical Chemistry

Cost analysis was accepted at the Barcelona conference as a special topic—this is the first time an international meeting has included the subject. The two major areas where cost analysis is urgently required in a medical laboratory which has an increasing lack of funds are in the discussion of strategies for selecting tests (which test, for example, has the highest discriminative power for a special diagnostic problem?), and in the choice of instruments (which instrument, or analytical system, produces the least costs for special diagnostic strategies?). Cost analysis is becoming more and more important to decision-making in medical laboratories.

Although the need for cost analysis appears to be generally accepted, it is still undecided as to who should collect and allocate costs and how this should be achieved. Comparison of costs can only be useful if the processes of collection and allocation are standardized and if definitions of terms used are internationally accepted—the language applied must be understood by all laboratory personnel.

The purpose of the symposium on cost analysis at Barcelona was to provide a general summary of the present status of cost analysis in the medical laboratory. Because of a limit on time, the reports presented could only introduce the field of costing. And the symposium was intended to stimulate interest in costing rather than to provide a detailed overview of the field.

The relation of the cost analysis symposium to automation (the primary topic of the Barcelona Congress) was evident: economical viewpoints are becoming more important, especially in terms of expensive instrumentation and large, automated multi-test systems which now influence the whole infrastructure of a medical laboratory.

The papers read at the symposium follow.

*Editor's note*: one symposium paper, received too late for this issue, will be published in *Journal of Automatic Chemistry*, Vol. 5, No. 3.

### Introduction

### Cost-benefit analysis

**R. Haeckel**

*Institut für Klinische Chemie, Medizinische Hochschule, Hannover, FR Germany*

The decision-making process surrounding the purchase of new instruments in a medical laboratory involves several steps, and many aspects have to be considered; this point was discussed during the cost-analysis symposium organized by the IFCC's Expert Panel on Instrumentation in collaboration with the IUPAC Commission on Automation and held at 1982's Barcelona meeting. The purpose of this paper is to discuss the economic aspects of the process. Because large analytical systems influence the whole organizational structure of the laboratory, cost analysis and cost-benefit analysis must be considered in respect of the total laboratory structure. The primary task of the medical laboratory is to produce test results—these usually lead to a number of effects:

Test<br>

↓←Expenditure<br>

Result– – →Efficiency<br>

↓<br>

Effect(s)– – →Effectiveness

The laboratory's efficiency in fulfilling this task can be measured by the quantity of results and by the costs incurred in obtaining them (expenditure). The laboratory's effectiveness is judged by its 'utility'. ('Utility' is a term commonly used in epidemiology and in social economics—see references [1–8].)

Costs are usually considered in monetary units, whereas utility can be monitored in monetary and in non-monetary units. Before costs are discussed in detail a few aspects of utility need to be considered.

Generally, an effective diagnostic strategy should lead to a gain in utility. The expense must, in some way, serve the patient, even if this is only a reduction in uncertainty [5]. The utility of diagnostic and therapeutic steps can be a reduction of disease course, a gain in life expectancy or prognostic hints. Utility can be studied on a micro- or macroeconomic scale. In the first case, utility is considered in a small unit (for example when buying a new instrument); in the second, utility is looked at on a much larger scale—its impact on a population for instance. If a negative test result leads to an unjustified therapy being followed or to an extension of a disease course, a loss of utility results. 'Net utility' is the sum of all positive and negative utilities.

Cost-utility investigations have been divided into cost-benefit and cost-effectiveness analyses [1–8] (see table 1).

In a cost-benefit analysis, expenses and utilities are considered in monetary units, these are finally summed so that the

